# Transforming X-ray detection with hybrid photon counting detectors

**DOI:** 10.1098/rsta.2018.0241

**Published:** 2019-04-29

**Authors:** Andreas Förster, Stefan Brandstetter, Clemens Schulze-Briese

**Affiliations:** DECTRIS Ltd, Täfernweg 1, 5405 Baden-Dättwil, Switzerland

**Keywords:** hybrid photon counting, macromolecular crystallography, X-rays, detector, synchrotron

## Abstract

Hybrid photon counting (HPC) detectors have radically transformed basic research at synchrotron light sources since 2006. They excel at X-ray diffraction applications in the energy range from 2 to 100 keV. The main reasons for their superiority are the direct detection of individual photons and the accurate determination of scattering and diffraction intensities over an extremely high dynamic range. The detectors were first adopted in macromolecular crystallography where they revolutionized data collection. They were soon also used for small-angle scattering, coherent scattering, powder X-ray diffraction, spectroscopy and increasingly high-energy applications. Here, we will briefly survey the history of HPC detectors, explain their technology and then show in detail how improved detection has transformed a wide range of experimental techniques. We will end with an outlook to the future, which will probably see HPC technology find even broader use, for example, in electron microscopy and medical applications.

This article is part of the theme issue ‘Fifty years of synchrotron science: achievements and opportunities’.

## 1. Introduction

Hybrid photon counting (HPC) detectors were shown to work at a synchrotron beamline in 1999 [1] and have since become standard at most synchrotron light sources [2,3]. They have dramatically improved X-ray detection over a wide energy spectrum and for a wide range of applications. Their success can be attributed to the accurate determination of the intensities of scattered photons over an extremely high dynamic range while ensuring single-photon sensitivity. Within a decade, most synchrotron beamlines for applications, such as protein crystallography, small-angle scattering, and surface and powder diffraction, have transitioned from charge-coupled device (CCD)-based detector systems and image plates to HPC detectors, which not only collect more accurate data than their predecessors but also offer opportunities for previously impossible research.

HPC detectors measure X-ray intensities by counting the photons incident on the active elements of the detector. The ‘hybrid’ in their name refers to their basic design principle. HPC detectors are made up of separately fabricated readout electronics and semiconductor sensors. During assembly of the detector, these elements are electrically connected with individual conductive bonds between each sensor and readout pixel.

From humble beginnings as derivative research projects at the Conseil Européen pour la Recherche Nucléaire (CERN) and the Paul Scherrer Institut (PSI), both in Switzerland, HPC detectors have been developed into superior technology. Current models come in a variety of sizes and shapes, are compatible with a number of different sensor materials optimized for different energy ranges, support dead-time-free data acquisition at high frame rates, detect up to 10 million photons per pixel and second with single-photon accuracy, and can be operated in vacuum.

Here, we will briefly survey the history of HPC technology from its origins in high-energy particle physics to first proofs of principle in X-ray detection and finally fully functioning detectors. We will clarify the aspects of the technology that are shared among all implementations and highlight important differences with their implications for experimental suitability. We will then show in detail how HPC detectors transformed first macromolecular crystallography and later a wide range of experimental techniques to become the detectors of choice for most synchrotron beamlines operating between 2 and 100 keV and other X-ray sources in the same range. We end by spelling out why we think HPC technology will find even broader use in the future, for example, in medical and industrial applications.

## 2. History

The predecessors of HPC detectors were hybrid pixel detectors that were developed for high-energy particle physics experiments at CERN in the 1980s [4,5]. These detectors were designed to characterize the particles created in high-energy collisions [6]. As these particles traverse the silicon sensor, they are not absorbed but leave behind a small amount of energy as a trajectory of ionization. This ionization charge is collected, amplified and quantified in the readout chip of the detector, which registers the position and arrival time of each ionizing particle as it traverses the sensor. By combining the information from several detective layers, the track of the ionizing particles can be reconstructed in three spatial dimensions and time.

A key innovation on the way to high-performance high-energy particle detectors was the separation of sensor and readout electronics. Particle sensors require highly resistive silicon, while the readout electronics require lower resistivity. By virtue of physically separating them, either could be optimized independently. In addition, the lower yield of the readout chips compared to the sensors can be compensated for by producing it in smaller sizes and tiling the back of each sensor with them. A sensor–chip hybrid is called a detector module. Any number of these rectangular modules can be combined to assemble larger detectors, with the size determined by the application.

The idea to develop an X-ray detector based on the principle of high-energy particles arose at CERN and at the PSI, which was contributing to the making of the detectors used at the Large Hadron Collider. Both detector groups realized that the silicon sensors used in particle detectors were perfectly suited to the detection of X-ray photons. The optimal readout circuitry has thus been the focus of intense development over the years. The 3 chip, developed at CERN, led to the first HPC detector tested with synchrotron radiation [1]. It was succeeded by various incarnations of the MEDIPIX chip [7]. At the PSI, this development was firmly with the application to macromolecular crystallography (MX) in mind.

By the mid-1990s, third-generation synchrotron light sources, such as the European Synchrotron Radiation Facility (ESRF) in Grenoble, France, the Advanced Photon Source near Chicago, USA, and SPring-8 in Harima, Japan, started to produce X-rays much too bright for existing detector technology to cope with. To get MX ready for the future, the construction of the first MX beamline at the Swiss Light Source (SLS) in Villigen, one of the first national third-generation synchrotron facilities, contained in its design concept the development of a single-photon counting hybrid pixel detector, which was recognized as the most suitable detector type for MX.

**Figure 1.**
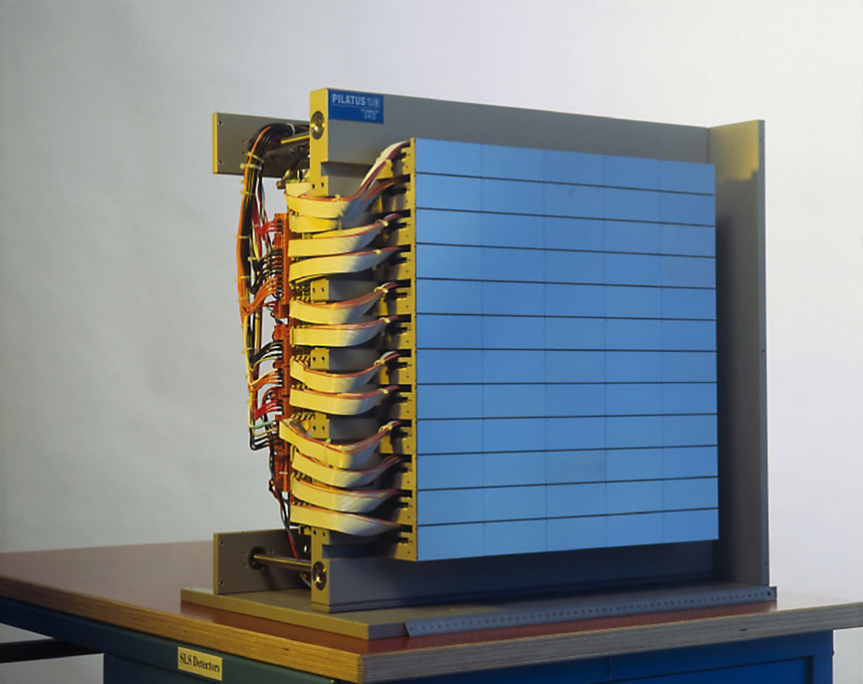
PILATUS 6M was the first large-area HPC detector in routine use at a synchrotron beamline. (Picture: Paul Scherrer Institute.) (Online version in colour.)

The first large-area HPC detector was built in 2003. Based on the experimental PILATUS readout chip, it had large pixels (217 × 217 µm^2^) and suffered from a large number of dead chips and non-responding pixels. Despite these disadvantages, many early experiments suggested that single-photon counting could provide diffraction data of superior quality [8]. Realizing this potential took a technological leap to the second generation of PILATUS [9]. The new readout electronics and smaller pixels (172 × 172 µm^2^) of the PILATUS2 chips were the basis of the first HPC detectors in routine beamline operation at any synchrotron (figure 1) [10]. In 2006, the PILATUS2 technology was commercialized by the company DECTRIS, which was spun out of the PSI for this purpose and has been highly successful ever since.

Data from the first HPC detectors presented considerable difficulties during processing. Because of the lack of dark current and readout noise, the algorithms in the data processing programs were not suitable to handle the extremely low background in the diffraction data. Getting the first program (XDS [11]) to successfully process what turned out to be excellent data took nearly a year. Once this problem was resolved, the advantages of HPC detectors (speed, dynamic range, point spread function and quantum efficiency) were widely accepted. By now, most MX beamlines have upgraded their equipment.

## 3. Technology

**Figure 2.**
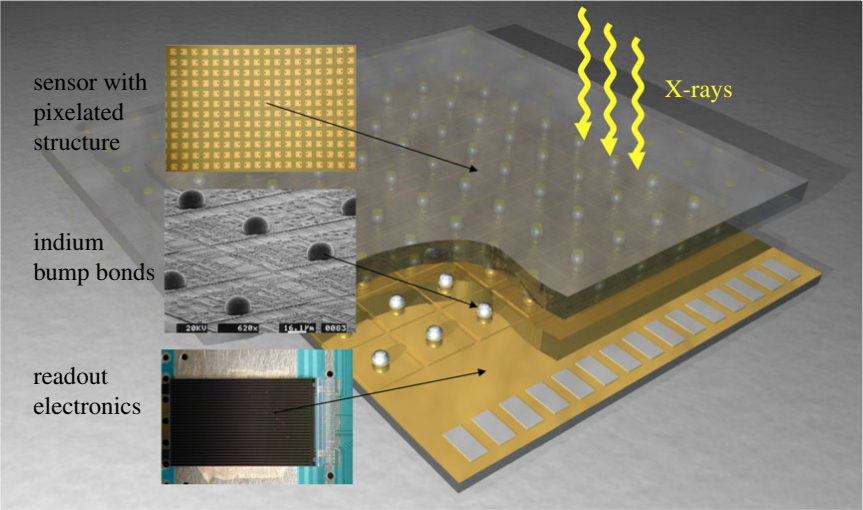
Schematic representation of a sensor readout hybrid as used in HPC detectors. Indium is not the only material used for the electrical connections between sensor and readout pixels. (Online version in colour.)

Figure 2 shows a simplified representation of an HPC pixel composed of sensor, readout electronics and an electrical connection between the two. HPC sensors are pixelated semiconductor crystals a few hundred micrometres thick. They absorb photons in an energy range determined by their material. Sensors made of doped high-resistivity silicon are most common. They efficiently absorb photons with energies between 2 and about 25 keV. At higher energy, the share of photons that pass through the sensor undetected increases. This can, in theory, be offset by longer exposure times, but many samples, in particular, biological materials, are easily damaged by radiation. This is why sensor materials with a higher stopping power, such as gallium arsenide (GaAs) and cadmium telluride (CdTe), have been developed for use with HPC detectors. Their high quantum efficiency extends the study of radiation-sensitive materials to energies up to 50 keV for GaAs and up to 100 keV for CdTe.

**Figure 3.**
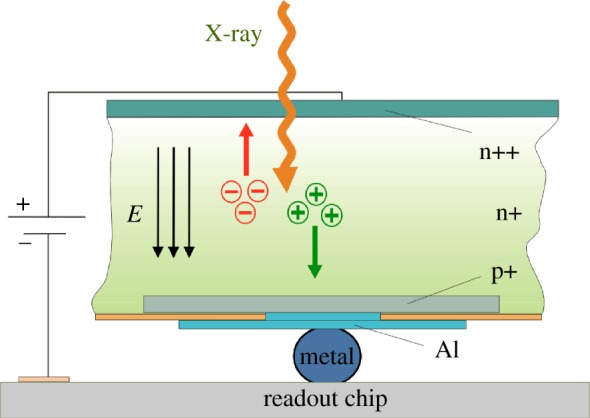
Side view of sensor readout hybrid. The polarity of the electric field (indicated by three parallel arrows) that separates the charge generated by the absorption of a photon depends on the requirements of the sensor material and the readout electronics. (Online version in colour.)

The absorption of an X-ray photon in the sensor generates a charge cloud made up of a number of charge carriers (electrons and holes) that is proportional to the energy of the absorbed photon. An electric field applied across the sensor drives the electrons (or holes, depending on the polarity of the field) towards an electrode at the bottom of the sensor (figure 3). Despite some diffusion and charge repulsion effects, the charge carriers stay close together and transfer the signal to the readout electronics with minimal point spread (a fraction of a pixel).

The lower the energy of the incident photons, the more difficult it is to detect them accurately. At energies lower than 2 keV, some photons might be absorbed in possible metallization layers or in the passivation layer of the sensor and avoid detection. In addition, the threshold is close to the electronic noise of the pixel electronics, which prevents a clear separation of the signal. Photons with higher energies than specified for a sensor cause different problems. They penetrate deeper, with the probability of traversing the sensor without being absorbed proportional to their energy. This causes a decrease in the quantum efficiency and eventually an unfitness of the sensor material for that photon energy. To avoid problems of radiation damage by penetrating photons, most readout chips are built according to radiation-tolerant design principles [12].

The readout chip is segmented into pixels the same size as the sensors. It is bonded to the sensor with small metal bumps (usually indium) that make electrical connections between corresponding pixels in the two layers and allow the charge induced in the sensor layer to be detected in the electronics. Normally, an array of several readout chips is bonded to each rectangular HPC sensor. These so-called modules, the largest of which cover around 8 × 4 cm^2^, carry between 100 000 and 750 000 pixels and are the building blocks of area detector assemblies. The modularity of the detector is an advantage when it comes to designing larger arrays or customized detector geometries.

Each readout pixel detects and amplifies the signals arriving from the corresponding pixel of the sensor layer. It increments a digital counter if the signal is higher than a preset threshold. The key advantage of using energy thresholds is to detect photons in a defined energy window. Set to somewhere between 40% and 80% of the photon energy, the threshold prevents the accumulation of electronic noise and dark current from the sensor and can suppress sample fluorescence if needed. Setting a second threshold to above the photon energy can help suppress higher harmonics, cosmic background and natural radioactive decay.

The digital storage of the number of counts detected avoids any readout noise and provides perfect linearity since no analogue-to-digital conversion step is involved. With a properly calibrated flat field that ensures equivalent responses from all pixels, the X-ray intensities can be measured with an accuracy limited only by the statistical uncertainty of the measurement (Poisson statistics). The counters can be addressed with row and column shift registers and read out in parallel [8]. This makes data acquisition at frame rates in the hundreds or thousands of hertz possible.

All counting detectors are count-rate-limited. Multiple coincident photons in one pixel will increment the counter by only one. Because of the time it takes to amplify and count a signal, there is a minimum delay until another photon can be detected after the first. For HPC detectors, this paralysis time is of the order of hundreds of nanoseconds and translates into a typical count-rate limit of 10^5^ to 10^7^ per pixel and second [13], which constitutes an improvement of several orders of magnitude with respect to earlier counting detectors, such as multi-wire and gas scintillation proportional counters. Algorithms that detect photons while the counter is still nominally paralysed can increase this limit further [14].

A number of different groups have designed counting readout electronics. The detector group at the PSI developed the original PILATUS and EIGER [15] chips. Detectors based on these chips and their successors are manufactured and sold by DECTRIS. The MEDIPIX collaboration (founded by CERN) has now developed three generations of their eponymous chip [16,17] and derivatives like TIMEPIX [18] and MEDIPIX3RX [19]. Detectors based on these chips are sold by a number of manufacturers, such as Advacam, Amsterdam Scientific, Quantum Detectors, MARS Bioimaging and X-Spectrum. The Italian National Institute of Nuclear Physics has developed three generations of PIXIE chips [20]. Malvern Panalytical now commercializes detectors based on this technology. The PXD18k chip developed at the AGH University of Science and Technology in Krakow, Poland, is the basis of detectors sold by Rigaku Oxford Diffraction [21].

## 4. Transforming macromolecular crystallography

In the mid-1990s, MX beamlines faced a number of challenges. The new third-generation synchrotrons provided unprecedented photon rates. Measuring diffracted photons accurately required a large dynamic range or short exposure times. Neither was possible or practicable with existing detector technology. CCD detectors were severely limited in their dynamic range and added substantial readout noise to each exposure. Image plates required readout times several orders of magnitude longer than exposure times, which led to the vast majority of photons provided during an experiment going to waste.

Another fundamental limitation of CCD and image plate detectors had to do with the method used to collect data. To cover more of reciprocal space, the sample is usually rotated while it is exposed to X-rays. At the end of a rotation increment, the exposure is stopped and the detector read out. A full dataset is assembled from images acquired at successive rotation increments, with the total rotation range large enough to ensure the measurement of each unique reflection. Rotation experiments can be described as widely or finely sliced, depending on whether the rotation increment is larger or smaller than the mosaicity of the crystal.

### (a) Fine slicing

Widely sliced data collection leads to fewer images with the intensities of most reflections measured fully on one image. Finely sliced data require many more images and most reflections are predominantly collected across several images as partials. On the upside, there will be fewer spatial overlaps, fewer saturated pixels and, in theory at least, lower X-ray noise because the amount of background noise recorded with each reflection is minimized, which leads to more accurate data [22]. In practice, fine slicing fails with CCD detectors because the detector contributes noise to every image, which counteracts the positive effect on data quality of lower background noise. With image plate detectors, fine slicing is impracticable because of long readout times per image.

HPC detectors promised to make full use of fine slicing [23]. With no detector noise and extremely short readout times, the design of experimental strategies would not be limited by hardware considerations any more. The first synchrotron beamline to experiment with and then use a large-area HPC detector was X06SA at SLS. Data collected with a first-generation PILATUS 1M established the potential for superior data quality despite serious issues with the build quality of the detector modules [8]. The successor to the PILATUS 1M was a prototype PILATUS 6M detector, based on the PILATUS2 chip [10]. This detector fundamentally changed how crystallographic experiments at synchrotrons were done.

**Figure 4.**
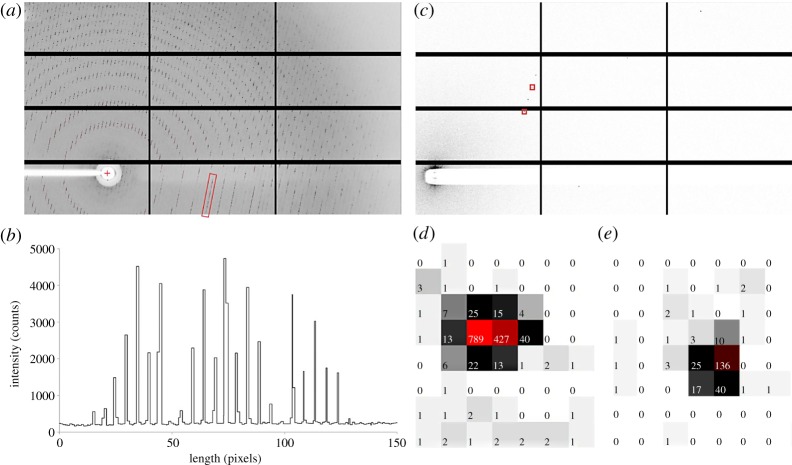
Example diffraction data. (*a*) Diffraction data from a 70S ribosome crystal. Only 12 out of 60 total detector modules are shown for clarity. A line plot along the reflections in the boxed region is shown in (*b*). Thanks to the direct detection of photons, adjacent peaks are clearly baseline-separated in spite of a largest unit cell dimension of 600 Å. Data were collected with a PILATUS3 6M detector at beamline 24-ID-C of the Advanced Photon Source (courtesy Yury Polikanov, University of Illinois at Chicago). (*c*) Diffraction data from a thaumatin crystal taken in vacuum. Only 12 of 120 total detector modules are shown for clarity. Two boxed reflections are magnified in (*d*) and (*e*). The lack of background noise results in exceptional signal-to-noise ratio. Data were collected with a PILATUS 12M detector on beamline I23 of Diamond Light Source (courtesy Armin Wagner, Diamond Light Source). (Online version in colour.)

Early experiments confirmed the benefits of finely sliced data [24]. They also helped exclude other sources of error. Instead of closing the shutter after each exposure for image readout, the shutter was left open throughout the dataset. This avoided shutter synchronization errors and increased goniometer accuracy. Shutterless data collection and fine slicing are now standard protocol. Instead of calculating a strategy to maximize reciprocal space coverage with the fewest images, users collect full 360° rotations at a low dose. As long as the permissible dose is not exceeded, this will maximize data quality and anomalous signal strength by maximizing multiplicity. This approach is particularly well suited to weakly diffracting crystals and led to the structure of the 80S ribosome [25]. For crystals with exceptionally large unit cells and low mosaicity, e.g. of the ribosome, an additional advantage of HPC detectors is the sharp point spread function that permits the separation of closely spaced diffraction spots (figure 4*a,b*).

Since the early days of HPC detectors, it has become normal for crystallographers to collect dozens of datasets per hour, watch the goniometer spin while the data are collected and see movies of collected data play on their screens in real time. The obtainable data quality is significantly higher than it was 15 years ago because of fine slicing, shutterless data collection, the lack of detector readout noise and sharper spots made possible by the direct detection of X-rays. A dataset's apparent resolution is higher because weak spots can be measured more accurately in the absence of noise. The adoption of HPC technology at synchrotrons has thus been nearly universal. More than 20 000 structures from data collected with HPC detectors have now been deposited in the Protein Data Bank, with a share of more than 50% of all new structures recently. At the same time, most other aspects of MX beamlines have also been improved, ushering in a golden era in crystallography.

### (b) Native SAD and high throughput

The higher data quality obtainable with HPC detectors revived interest in experimental phasing by single anomalous wavelength dispersion (SAD) [26]. It also made it possible to design arbitrarily complex experiments to acquire anomalous signal sufficient for phasing even from samples with only weak native scatterers simply by collecting more data [27]. With no detector noise, the signal will continue to increase until it hits a ceiling set by the systematic error of the experiment. For native SAD strategies to be successful, the dose per image must be low enough not to exceed the total permissible dose for the sample. This so-called dose fractionation is possible only with detectors without readout noise. Putting sample and detector into vacuum further decreases background noise (figure 4*c–e*) and increases data quality. In addition, the use of longer wavelengths made possible by the lack of absorption in vacuum brings most elements in the periodic table within reach of single- or even multiple-wavelength anomalous dispersion experiments [28].

The high frame rate of HPC detectors also increased the throughput of MX beamlines. This again changed the way crystallographers work. Instead of screening crystals in the laboratory and taking the best to the synchrotron, screening is now performed at the synchrotron. Pharmaceutical companies see much higher value in synchrotrons now that they can get of the order of 100 datasets from an overnight shift. Beamline I04-1 at the Diamond Light Source has taken this concept to its culmination. By automating nearly all of the steps from the soaking and harvesting of crystals until data analysis, a ligand screening campaign with 1000 compounds can be completed within one week [29].

Together with beamline automation and faster goniometers, high-speed HPC detectors have enabled a number of methods that depend on the collection of a large number of images. Grid scanning in two dimensions makes crystal centring by X-ray diffraction possible. This avoids possible lens effects with optical microscopy and ensures that the crystal stays in the beam throughout data collection. It is at the heart of the fully automated beamlines such as MASSIF-1 at ESRF [30] that provide data of exceptional quality without user intervention [31]. Diffraction cartography allows the identification of multiple tiny crystals [32] or crystals grown in lipidic cubic phase [33] that would otherwise remain invisible and opens the door to serial crystallography. With helical scanning data collection, the entire volume of elongated and needle-shaped crystals can be used for diffraction [34].

### (c) Serial and time-resolved crystallography

Serial crystallography was first developed at X-ray free electron lasers (XFELs) where structures can be determined from individual diffraction images collected on thousands or hundreds of thousands of microcrystals [35]. Fast HPC detectors and beamline automation have brought the ideas behind MX at XFELs to synchrotrons [36,37]. Supplying crystals on solid supports, such as chips or grids, instead of in jets or streams dramatically improves data collection efficiency [38–41]. Serial crystallography can be done in a way to obtain time-resolved information on the order of milliseconds [42]. This helps shed light on important biological processes, especially given that most work is done at room temperature.

MX would be almost unrecognizable to a practitioner from 15 years ago when flux limitations caused long exposure times, and image plates and CCDs required shutter closures and long readout times after each image. The sweeps of data collected in a shutterless mode within a few seconds are the closest to this earlier way of collecting data, but the achievable quality is much higher. In other words, much weaker diffracting crystals can now be studied by MX. Serial crystallography has turned MX into a different game altogether. In the near future, most technical and methodological development will probably focus on this area, and crystal quality will become the ultimate limitation of future MX methods.

## 5. Transforming X-ray sciences

While the impact of HPC detectors on MX has been most visible, the benefits of the technology, such as adjustable threshold, low noise, high frame rates and high count rates, have been advantageous in many other fields of research at synchrotrons and in laboratory X-ray facilities as well. Some examples are presented below. This selection is necessarily incomplete.

### (a) Plasma spectroscopy

Hot plasmas confined within magnetic fields are important in nuclear fusion and astrophysical plasma research. The study of the low-energy radiation (1.6–6 keV) these plasmas emit is important for their understanding. Specifically, from measurements of plasma X-ray emissivity at high spatial and spectral resolutions, the core electron temperature of the plasma and impurity density can be calculated accurately. Because of their high frame rates, adjustable thresholds and large uniform surface, HPC detectors are well suited to this application, and specific calibrations further improve analysis methods [43]. The achievable threshold energy resolution is of the order of 100 eV even at the lowest energies [44].

### (b) X-ray scattering

The field of X-ray scattering is vast but the goal is always the understanding of the nanostructure of large amounts of the non-crystalline sample at the mesoscopic scale. Small-angle X-ray scattering (SAXS) studies structures at the nanometre range. Wide-angle X-ray scattering (WAXS) yields information at the atomic scale. Both rely on very low background noise, a high count rate and a low point spread function to resolve adjacent signals (which might be of vastly different magnitude) [45]. For maximum spatial resolution, multiple detector systems can be combined, which makes simultaneous SAXS and WAXS measurements possible [46]. Spurious background from windows and air can be efficiently eliminated by operating the detectors in vacuum.

Grazing-incidence SAXS is used to study the surface properties of materials [47]. A variant, critical-dimension SAXS, has recently been developed to monitor the feature size and uniformity of repetitive features smaller than 10 nm on silicon chips [48]. With their high frame rates and high signal-to-noise ratio, HPC detectors enable data acquisition for reliable quality control during fabrication in the semiconductor industry.

An exciting recent development has been SAXS–computed tomography. Here, a solid sample, such as a bone or tooth, is scanned and rotated while scattering data are collected. These data are then computationally analysed for a multi-scale, tensorial understanding of the material properties of the sample. The technique depends on high-flux synchrotron beamlines, fast, noise-free HPC detectors and vast computing power but can give otherwise inaccessible information on the nanostructure of macroscopic specimens [49,50].

### (c) Diffuse scattering

Besides the Bragg peaks used to solve static structures of proteins and small molecules, X-ray diffraction patterns include diffuse intensities arising from departures from the ideal crystal lattice [51]. Measuring these intensities accurately yields information on collective motions in crystals. The high dynamic range, low noise and low point spread have made HPC detectors the best tools to measure diffuse scatter, which is extremely weak and in the vicinity of strong diffraction peaks [52]. Efficient suppression of fluorescence by means of the low energy threshold further improves the detection of weak diffuse features.

### (d) Chemical crystallography

Crystallography of non-biological materials with unit cells usually smaller than 50 Å in the largest dimension was slow to adopt HPC detectors. This was probably due to the generally strong reflections that made signal-to-noise considerations less pressing than in MX. By now, specialized beamlines at synchrotrons operate with HPC detectors [53] and a large share of laboratory systems are sold with HPC detectors as well.

The key benefit of chemical crystallography is fluorescence suppression. Fluorescence increases the background noise and thus decreases the achievable accuracy of the data. The adjustable threshold can be set to above the energy of the fluorescence, which is then not detected. This leads to much more accurate data and better structures.

Thanks to the high frame rate of HPC detectors, data can sometimes be acquired quickly enough to study chemical reactions *in situ*. For example, an electrochemical cell was used at BM01 at the ESRF to study the oxidation of strontium ferrite [54]. This marked the first time a complete solid-state reaction was investigated by X-ray crystallography under *in situ* conditions.

The minimal background noise of HPC detectors extends the resolution at which the intensities of reflections can be measured with statistical significance. Accurate high-resolution information results in electron density maps with enough detail to study chemical bonds and electronic configurations. Together with quantum mechanical calculations, this allows one to go beyond the spherical-atom approximation of X-ray crystallography and get a correct picture of the atomic structure of materials [55].

### (e) Powder X-ray diffraction

With HPC detectors, entire diffraction patterns can be acquired at a quality previously only available from point detectors. This has allowed powder X-ray diffraction (PXRD) to address formerly inaccessible questions. Some examples are the characterization of semi- and non-crystalline materials, the *in situ* monitoring of structural phase transitions upon heating or increases in pressure, and spatially resolved X-ray diffraction. The first HPC strip detectors were already combined into large linear assemblies to increase the accessible *q* range and shorten experiment times by doing away with scans [56].

As with chemical crystallography, PXRD can be done at high-flux beamlines with fast HPC detectors to study chemical reactions *in situ*. For example, ball mills placed in the beam allow one to study phase transitions and polymorphs in solid-state reactions and to observe intermediates that might not be accessible in solution [57]. Obvious applications for such studies are the development of hydrogen storage and battery technologies.

### (f) X-ray spectroscopy

Several early papers report on the successful use of HPC detectors for time-resolved X-ray absorption fine structure (XAFS [58]), X-ray photon correlation spectroscopy (XPCS [59]), high-resolution X-ray fluorescence (XRF [60]) and X-ray absorption spectroscopy (XAS [61]) studies but this has not become routine until recently. Now, XRF and XAS set-ups based on HPC strip detectors and wavelength-dispersive crystals have been developed for the laboratory [62,63]. At the synchrotron, extended XAFS studies have been done to simultaneously investigate surfaces and buried interfaces in multi-layered samples [64].

### (g) High-energy applications

The ready availability of high-energy X-rays at modern synchrotrons has aided the development of techniques, such as total scattering, *in situ* PXRD and X-ray diffraction–computed tomography (XRD-CT), for the time- and spatially resolved study of materials with various degrees of crystallinity. With image plates or flat panels, decent data could be collected in seconds to minutes, but improving data quality and decreasing exposure times remained elusive [65].

HPC detectors with CdTe sensors are now in use at several synchrotrons, where the advantages and challenges are becoming clear [66]. Initial problems with polarization have been overcome and the sensor quality of CdTe is approaching that of silicon. With accurate flat fields collected at the settings of the experiments, even high-quality data sufficient for pair distribution function analysis can now be measured.

## 6. Outlook

After transforming research at synchrotrons so comprehensively, what is the future of HPC detectors? We see four fields where HPC detectors are currently not used to detect X-rays and discuss their promise to transform measurements.

HPC is the logical choice for medical applications. On a very basic level, the negligible intrinsic background makes X-ray imaging safer by reducing the dose required for a certain image quality. In addition, detectors with multiple thresholds make spectral imaging possible. Multiple images with different energy cut-offs can be collected simultaneously, which results in additional information of diagnostic value with the same dose [67]. To be successful in medical imaging, HPC detectors need to be redesigned to have smaller or no gaps between modules. For use in CT, this caveat does not apply to the same extent, as gaps can be interpolated during scans, especially if many images are acquired quickly. It is likely that medical HPC detectors will thus be used in CT [68].

In industry, materials and products are often inspected and tested with X-rays. The characterization of residual stress of metal parts is important to detect fatigue before failure [69]. Different types of plastics need to be separated to ensure effective recycling [70]. The acquisition of images in up to four energy bands in a single exposure enables spectral methods such as material identification [71]. The high speed of data acquisition of HPC detectors is a prerequisite for their use in production facilities.

XFELs are one of the few fields of X-ray research where HPC detectors are unsuitable. As the counting of each photon takes a few dozen to a few hundred nanoseconds, the femtosecond pulses produced by XFELs cannot be resolved with HPC detectors. Hybrid detectors based on integrating readout electronics [72–74] will bring the high spatial resolution and high quantum efficiency made possible by direct detection to XFELs.

The next generation of synchrotrons, diffraction-limited storage rings, will deliver photon fluxes far beyond what is available now. At first glance, integrating detectors seem a more obvious choice than HPC detectors for new beamlines. However, while integrating detectors can detect photons arriving concurrently (i.e. have no instantaneous rate limit), they are limited in the amount of charge they can integrate. The latest HPC detectors offer comparable sustained photon detection rates as integrating detectors operated up to 1 kHz. Which technology is better will depend on the nature of the experiment [75].

Among other technical advances, direct electron detectors (DEDs) have transformed electron microscopy, in particular, single-particle analysis, over the last few years [76,77]. With the bottleneck of crystallization removed, biologically interesting structures are increasingly being determined by electron microscopy. Even conformationally heterogeneous samples can now be studied structurally at high resolution [78]. However, despite their improvements over existing technologies, DEDs fall short of addressing all requirements of electron detectors, namely speed, accuracy and dynamic range [79]. HPC detectors combine these three features and can detect electrons. They have already been used successfully in electron diffraction experiments [80,81]. Because of the stronger interaction of electrons with matter, good data can be collected from crystals isolated from crystalline powders by microscopic imaging. This promises to change how the pharmaceutical industry evaluates synthesized drug candidates. A broader applicability of HPC detectors in transmission electron microscopy and scanning electron microscopy is dependent on specifically designed readout electronics and possibly smaller pixels.

HPC detectors have transformed most applications at synchrotron light sources and have opened new opportunities in laboratory research. The potential for applying their transformative effect to related fields like medical imaging, industrial X-ray application and electron microscopy is large. The story that started more than 20 years ago with research into the physics of high-energy particles, nominally a purely academic undertaking, continues to make an impact in an expanding range of fields.

## Data accessibility

This article has no additional data.
